# Elucidate the quantitative early diagnostic indicators of mild chondromalacia patella: ratios of trochlear sulcus angle to patellar and trochlear widths

**DOI:** 10.3389/fmed.2026.1697996

**Published:** 2026-07-02

**Authors:** Haoxian Wang, Zhian Chen, Zijian Cao, Jie Xiao, Yun Zheng, LuanXing Yi, Neng Mao, Hongbo Tan, Xuesong Han

**Affiliations:** 1 Department of Limb Reconstruction, Fuzhou Second General Hospital, Fujian Provincial Clinical Medical Research Center for First Aid and Rehabilitation in Orthopedic Trauma, School of Clinical Medicine of Fujian Medical University, Fuzhou, Fujian, China; 2 Department of Orthopedics, Fuzong Clinical Medical College of Fujian Medical University, Fuzhou, China; 3 Department of Orthopaedics, People’s Liberation Army Joint Logistic Support Force 920th Hospital, Kunming, Yunnan, China; 4 Department of Orthopedics, The 900th Hospital of Joint Logistic Support Force, PLA, Fuzhou, China

**Keywords:** chondromalacia patella, early diagnostic indicators, elucidate, patellofemoral engagement, trochlear sulcus angle

## Abstract

**Background:**

Chondromalacia patella (CMP) is often exacerbated by anatomical variations in patellofemoral engagement, and there are currently no sensitivity early diagnostic indicators for mild CMP. This study focused on the relationship between normal patellofemoral joint parameters and mild CMP, aiming to provide a more accurate scientific basis for the early diagnosis and treatment of CMP by studying new quantitative ratios as early diagnostic indicators.

**Methods:**

The study involved 100 patients with mild CMP (modified Noyes grade 1 and 2A) and 100 healthy volunteers (controls) who underwent imaging from 1/4/2023 to 1/4/2024. Magnetic resonance imaging and T2 mapping were performed to measure the trochlear sulcus angle (TSA), lateral trochlear inclination (LTI), trochlear depth (TD), trochlear width (TW), patellar width (PW), trochlear length(TL), patellar length(PL), patella-patellar tendon angle(P-PTA), and quadriceps-patellar tendon angle(Q-PA). Differences in demographics and all measurements between the mild CMP group and the control group were detected. Novel composite ratios integrating trochlear morphology and patellofemoral engagement were established for mild CMP diagnosis. Univariate and multivariate logistic regression (adjusted for sex, age, side, height, weight, BMI) identified significant predictors. Receiver operating characteristic (ROC) curve of the participants was analyzed to describe the accuracy of each diagnostic test using the area under the curve (AUC). The ability of TSA, LTI, TD, and six different ratios to predict mild CMP was evaluated by calculating the odds ratio (OR), sensitivity, specificity, positive predictive value (PPV), and negative predictive value (NPV) for each measurement.

**Results:**

There was no significant difference in demographic characteristics between the two groups. Patients with mild CMP exhibited notable alterations in trochlear morphology and patellofemoral engagement parameters compared with controls. Among the evaluated imaging indicators, the composite ratios integrating trochlear sulcus angle with patellar and trochlear morphology demonstrated superior performance for the early identification of mild CMP. After adjustment for demographic variables, the ratio of trochlear sulcus angle to trochlear width (TSA/TW) remained the most robust independent diagnostic factor. Furthermore, ROC analysis demonstrated that TSA/TW provided the highest diagnostic accuracy and outperformed traditional imaging thresholds and other composite ratios in distinguishing mild CMP.

**Conclusion:**

The composite ratio TSA/TW is a potent independent early diagnostic factor of mild CMP, demonstrating superior diagnostic accuracy over traditional methods This ratio-based approach shows significant potential to improve early diagnosis of patellofemoral degeneration.

## 1 Introduction

Chondromalacia patella (CMP) is a degenerative disease of the patellar cartilage (1). The prevalence of CMP in the general population is as high as 36.2%, but it may reach 50.0% among middle-aged patients aged 30–40 years (2). Factors such as fractures, dislocations, rheumatic diseases, and insufficient blood supply often play a role in the etiology of CMP, and many affected patients also have varying degrees of patellofemoral joint dyskinesia and/or abnormal patellar movement trajectories (3, 4). These abnormalities cause long-term high pressure on the lateral patella (5), leading to the release of inflammatory factors and causing patellofemoral joint pain disease progression. In the early stages (grades 1 and 2), CMP has the ability to self-repair (6). However, in the late stages (grades 3 and 4), the cartilage lesions in CMP have progressed to osteoarthritis of the patellofemoral joint (7–9). Therefore, early diagnosis and treatment of CMP are crucial. Arthroscopy is the most effective evaluation method for diagnosing CMP; however, this technique is invasive. With the progress of imaging technology, magnetic resonance imaging (MRI) has gradually replaced arthroscopy as the most commonly used examination method for clinical diagnosis of CMP (6, 10). MRI can clearly show the arrangement of the patellofemoral cartilage, bone, soft tissue, and patellofemoral joint. However, the reported diagnostic performance of fat-suppressed MRI sequences (e.g., axial T1-weighted spin-echo, T1 fat-saturation, and proton density–weighted imaging) for detecting early CMP-related cartilage lesions remains highly variable. Early studies reported sensitivities ranging from 2% to 100%, specificities from 50 to 94%, and overall diagnostic accuracies of 77–90% (11–13).

With advances in MRI technology, including the widespread adoption of 3.0-T scanners and the development of quantitative techniques such as T2 mapping, recent studies (2010–2024) have demonstrated improved diagnostic performance for mild CMP. Compared with conventional morphological MRI assessment, T2 mapping is more sensitive in detecting early cartilage degeneration before visible structural damage occurs, thereby emerging as a valuable imaging tool for the quantitative early diagnosis of mild CMP and the identification of subtle patellofemoral cartilage abnormalities. Zagaria et al. (7) reported a sensitivity of 72% and a specificity of 85% for early patellofemoral chondropathy using T2 mapping. Ambra et al. (8) showed that MRI-based anatomical parameters, such as trochlear depth, achieved a diagnostic accuracy of 78% for focal patellar cartilage lesions. Furthermore, Tanaka et al. (14) confirmed that quantitative trochlear parameters, including the trochlear sulcus angle (TSA), enhanced the diagnostic efficiency of early trochlear dysplasia—a key predisposing factor for CMP—with an area under the curve (AUC) of 0.76. Despite these technological advances, several important limitations persist. First, early-stage CMP (modified Noyes grade 1) often lacks overt morphological changes on conventional MRI, resulting in limited sensitivity. Second, inter-observer variability in the interpretation of cartilage signal alterations remains non-negligible (15, 16). Third, reliance on absolute values of single parameters (e.g., TSA) does not adequately account for inter-individual differences in patellofemoral anatomical congruence (17).

Research has shown that a flat and shallow trochlea (high sulcus angle) is associated with patellar softening of the cartilage (18–20). Traditionally, the Dejour classification has been used to assess the severity of trochlear dysplasia based on the appearance of the trochlea on lateral X-ray images of the knee (21, 22). However, there are significant differences in the reliability of the Dejour classification when using different imaging techniques (16, 23). Therefore, scholars have successively conducted relevant studies to quantify indicators of trochlear dysplasia, including the trochlear sulcus angle (TSA), lateral trochlear inclination (LTI), and trochlear depth (TD). Tanaka et al. (14) used Youden’s J index and the area under the curve (AUC) under T2-weighted MRI to calculate a TSA of >145°, LTI of <17°, and TD of <4 mm as the optimal thresholds for defining trochlear dysplasia. TSA is the best indicator for early identification severe CMP, but its sensitivity for diagnosing mild CMP is poor (10). Additionally, as an absolute value, TSA does not take into account the patient’s trochlear anatomy and patellofemoral engagement. Previous studies have suggested that an increase in the patellofemoral volume ratio and patellar width (PW) to trochlear width (TW) ratios may lead to improper patellar engagement within the femoral trochlea (17, 24–26). TSA/TW essentially represents the trochlear groove opening angle per unit trochlear width, whereas TSA/PW represents the trochlear groove opening angle per unit patellar width, respectively, and quantify the relative restraining capacity of the trochlea on the patella. Higher ratios indicate reduced lateral containment, which may lead to abnormal patellar tracking and increased localized contact stress, thereby promoting early cartilage degeneration (17, 26–28).Therefore, the combination of trochlear morphology and engagement parameters as a novel strategy to improve diagnostic sensitivity.

This study was performed to establish a more accurate predictive ratio for mild CMP by associating the trochlear shape with patellofemoral engagement parameters, thereby addressing the limitations overcoming the limitations of using trochlear shape or patellofemoral joint parameters alone for diagnosing mild CMP.

## 2 Materials and methods

### 2.1 Experimental design

This study was approved by the Institutional Ethics Committee of the 900th Hospital of the Joint Logistic Support Force (Approval No. Ethics 2023-089), and all procedures were conducted in accordance with relevant ethical guidelines and regulations. The experiments informed consent to participate was obtained from all of the participants in the study and informed consent to participate was obtained from the parents or legal guardians of any participant under the age of 16. Using the electronic medical record system of the XXBLINDEDXX, we identified patients aged 15–40 years with mild CMP (modified Noyes grade 1 and 2A) who had experienced knee pain for >3 months and undergone knee MRI and quantitative T2 mapping from 1/4/2023 to 1/4/2024. Patients with severe patellar cartilage injury, a history of knee joint surgery, trauma, and clinical signs or imaging findings of joint stenosis, osteophytes, tendonitis, extensive knee joint effusion, inflammatory arthritis, advanced osteoarthritis, knee joint deformity, or space-occupying masses were excluded from this study. Additionally, this study excluded patients with acute patellar dislocation or severe trochlear dysplasia (Dejour types B–D), enrolling only those with mild trochlear dysplasia (Dejour type A) or normal anatomical structure to ensure that the findings more accurately reflect the pathological mechanism of early-stage CMP. In total, 100 patients with CMP were analyzed.

The sample size was calculated using G*Power 3.1 software based on the primary outcome indicator (TSA/TW ratio). Referring to previous studies on trochlear morphological parameters and CMP (14, 29), we assumed a medium-to-large effect size (Cohenchle = 0.7), α = 0.05, and statistical power (1-β) = 0.9. The calculated minimum sample size per group was 86. Considering potential dropout or exclusion of invalid data, we enrolled 100 patients in the mild CMP group and 100 healthy controls to ensure sufficient statistical power for detecting significant differences between groups.

**FIGURE 1:**
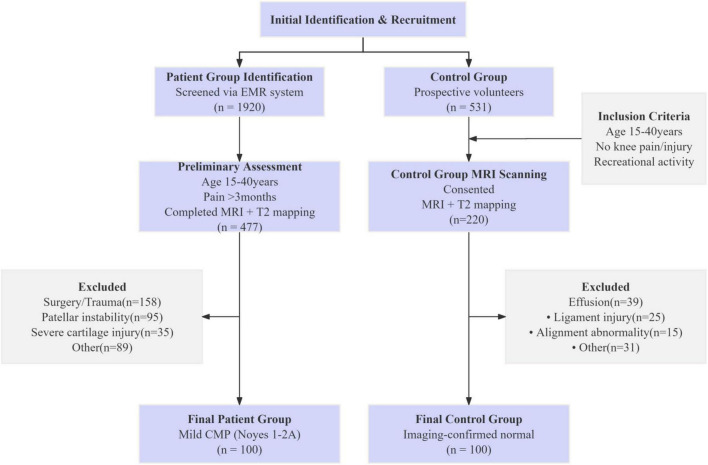
Flowchart of participant screening and enrollment for the study. CMP, chondromalacia patella; EMR, electronic medical record.

Healthy controls were prospectively recruited from our health examination center database, internal bulletins, and social media. Potential volunteers were screened via a structured telephone interview. Preliminary inclusion criteria were: age 15–40 years, no history of knee pain, significant injury, or surgery, and a recreational (non-competitive) activity level. Eligible individuals provided informed consent and underwent the same unilateral MRI and T2 mapping protocol as the patient group. Only those with imaging-confirmed absence of abnormal lower limb force lines, knee joint effusion, advanced osteoarthritis, knee joint deformities, space-occupying masses, fractures, or signs of previously undiagnosed ligament injuries were included (Figure 1).

### 2.2 Imaging evaluation

The MRI was performed using a 3.0T scanner (uMR 770; United Imaging Healthcare, Shanghai, China) with a dedicated 12-channel transmit/receive knee coil. To optimize patellofemoral joint assessment, all patients were positioned supine with the affected knee flexed at approximately 30° and stabilized within the coil. A standardized imaging protocol was used for all participants without variation throughout the study period to ensure consistency and reproducibility. The specific parameters for each sequence were as follows:

Conventional Morphological Sequences:

- Sagittal rapid spin-echo T1-weighted: repetition time (TR)/echo time (TE), 560/9.02 ms; matrix, 320 × 272; field of view (FOV), 18 × 18 cm; slice thickness, 3.5 mm
- Sagittal fat-suppression proton density-weighted: TR/TE, 2,592/38 ms; matrix, 304 × 258; FOV, 18 × 18 cm; slice thickness, 3.5 mm
- Axial rapid spin-echo T1-weighted: TR/TE, 520/8.96 ms; matrix, 320 × 256; FOV, 16 × 16 cm; slice thickness, 3.5 mm.
- Axial fat-suppression proton density-weighted: TR/TE, 2,500/45.76 ms; matrix, 320 × 240; FOV, 16 × 16 cm; slice thickness, 4.0 mm
- Coronary fat-suppression fast spin-echo T2-weighted: TR/TE, 2,020/38–64 ms; matrix, 228 × 228; FOV, 16 × 16 cm; slice thickness, 3.5 mm
- Axial fast spin-echo T2-weighted: TR/TE, 5,545/85.92 ms; matrix, 272 × 217; FOV, 18 × 18 cm; slice thickness, 3 mm

Quantitative T2 Mapping Sequences: o

- Sagittal T2 mapping: TR/TE, 2,040/14.64–73.2 ms; matrix, 304 × 228; FOV, 16 × 16 cm; slice thickness, 3.4 mm
- Axial T2 mapping: TR/TE, 2,097/15–75 ms; matrix, 288 × 216; FOV, 14 × 14 cm; slice thickness, 4 mm

**FIGURE 2:**
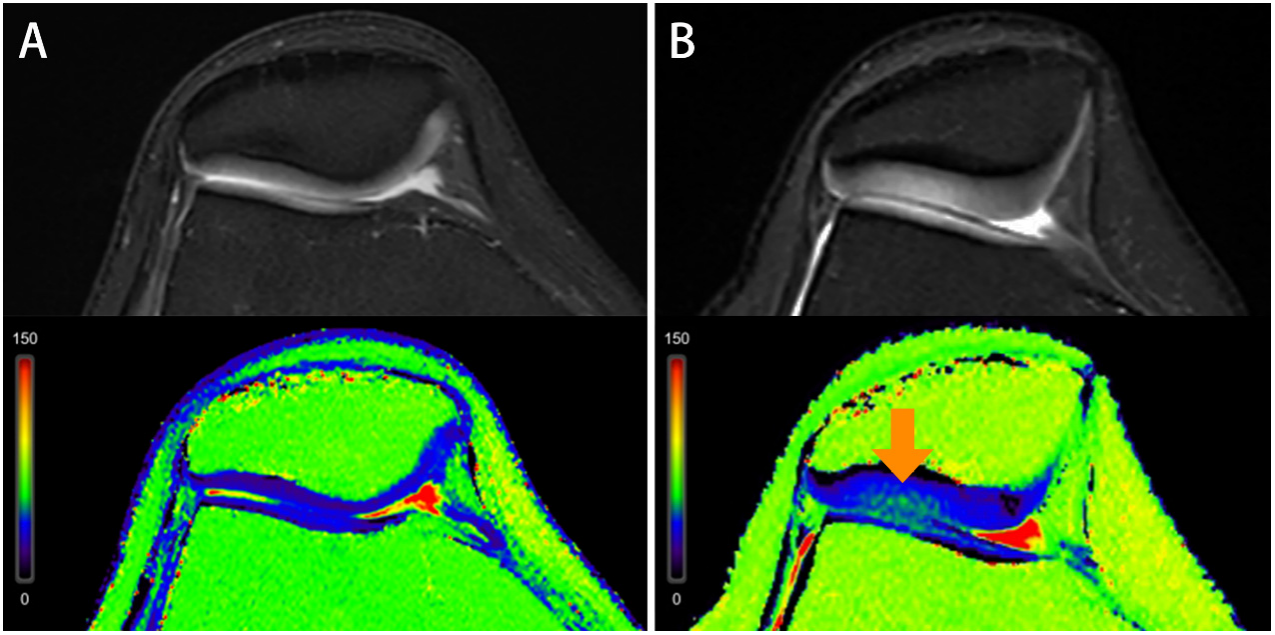
Evaluation of T2 mapping of patellofemoral joint cartilage. **(A)** The fat-suppressed proton density images and corresponding T2 mapping images of normal patellar cartilage showed a shorter T2 relaxation time. **(B)** The fat-suppressed proton density images of patients with CMP showed no significant cartilage damage, while the corresponding T2 mapping images showed an increase in the T2 relaxation time. The arrows indicate modified Noyes grade 1 CMP.

Each patient’s patellar cartilage was evaluated using fat-suppressed proton density-weighted imaging and corresponding T2 mapping. To maintain consistency in the evaluations, patellofemoral cartilage injuries were evaluated by a sports medicine physician with 20 years of experience using the modified Noyes grading system (Figure 2). This system categorizes cartilage damage into the following grades: 0, normal cartilage; 1, increased T2 signal intensity in cartilage with normal morphology; 2A, surface partial-thickness cartilage injury involving <50% of the total thickness; 2B, deep partial-thickness cartilage injury involving >50% of the total thickness; and 3, full-thickness cartilage injury. If different grades of cartilage damage were present, the most severe grade was used as the basis for evaluating the severity of patellar cartilage involvement. In the statistical analysis, grade 0 was classified as the normal group, while grades 1 and 2A were classified as the mild CMP group (10, 26, 30).

### 2.3 Anatomical measurement

In addition to TSA, LTI, and TD, the following anatomical measurements of the patellar joint were also obtained by MRI: TW in the axial position, PW in the axial position, trochlear length (TL) in the sagittal position, patellar length (PL) in the sagittal position, patella-patellar tendon angle (PPTA) in the sagittal position, and quadriceps-patellar tendon angle (QPA) in the sagittal position. The measurement results were obtained and recorded by three trained sports medicine physicians. The image evaluation employed a triple-blinding design: (1) evaluating physicians were blinded to each other’s measurement results; (2) physicians were blinded to patient group assignment (case/control group); (3) data analysts were not involved in the image interpretation process. To calculate the intra-rater reliability of a single observer, the observer conducted three measurements on 10 randomly selected knees, with a 3-h interval between each measurement. The reliability between evaluators and within evaluators was demonstrated with the INFINITT PACS system, version 3.0.11.3 (Healthcare Information Technology, Seoul, South Korea) and previously validated techniques, using both blind and random methods. All comparisons were made using the average measurement of the three observers.

This study established novel composite ratios by integrating trochlear morphological parameters with measures of patellofemoral engagement, providing an assessment system with enhanced sensitivity for the prediction of mild CMP. For this purpose, we constructed a predictive model using the ratio of TSA to six patellofemoral parameters (TW, PW, TL, PL, PPTA, and QPA). These six ratios were TSA/TW, TSA/PW, TSA/TL, TSA/PL, TSA/PPTA, and TSA/QPA. The objective of this study was to identify the anatomical ratio and its optimal critical value that can maximize the prediction of CMP.

To optimize visualization accuracy, measurements were performed on specific MRI sequences based on the anatomical structures of interest.

#### 2.3.1 Axial measurements

TSA, LTI, and TD were measured on axial T1-weighted images to ensure clear visualization of bony landmarks. Following the protocol established by Tanaka et al. (14), these three parameters were measured on the same single axial slice, defined as the most proximal image containing complete cartilage coverage of the trochlea. This specific level was selected to characterize the morphology of the proximal trochlea, where manifestations of dysplasia are most pronounced.

**FIGURE 3:**
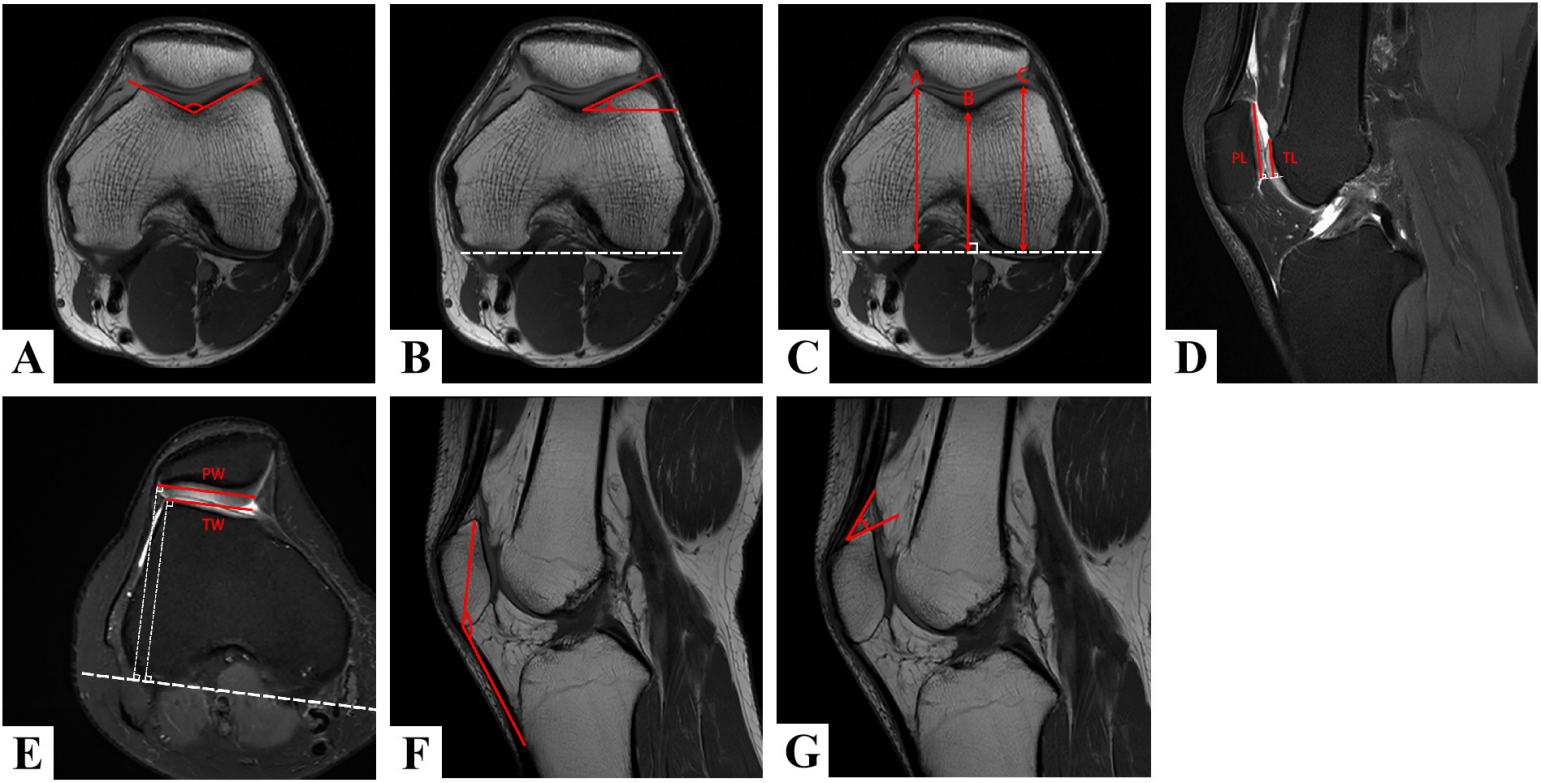
Anatomical measurements of patellofemoral engagement. **(A)** TSA, trochlear sulcus angle. **(B)** LTI, lateral trochlear inclination. **(C)** TD, trochlear depth. **(D)** TL, trochlear length and PL, patellar length. **(E)** TW, trochlear width and PW, patellar width. **(F)** P-PTA, patella-patellar tendon angle. **(G)** Q-PA, quadriceps-patellar tendon angle.

- TSA was defined as the angle formed by the line connecting the deepest part of the trochlear groove to the most protruding part of the lateral condyle and the line connecting the deepest part of the trochlear groove to the most protruding part of the medial condyle (Figure 3A).
- LTI was measured based on the description by Carrillon et al. (31) as the angle between the posterior condylar line and the line along the lateral surface of the trochlea (Figure 3B).
- TD was defined as the distance from the posterior condyle line to the most prominent points of the lateral condyle, trochlear groove, and medial condyle (Figure 3C). The depth was calculated according to the method described by Pfirrmann et al. (32).Specifically, subchondral bone landmarks were utilized to assess the underlying osseous morphology, thereby avoiding inaccuracies caused by variable cartilage thinning associated with CMP.

In contrast, TW and PW were measured on axial fat-suppression PD images to clearly define cartilage boundaries. According to Guilbert et al. (33), these measurements utilized two different axial levels: the slice demonstrating the widest lateral trochlear border (for TW) and the slice showing the widest patella (for PW).

- PW was defined as the maximum medial-to-lateral distance of the patellofemoral joint surface, measured parallel to the tangent line of the femoral posterior condyle.
- TW was defined as the distance from the inner side of the patellar joint cartilage to the outermost side of the trochlear cartilage (Figure 3E).

#### 2.3.2 Sagittal measurements

PL and TL were measured on sagittal fat-suppression PD images to evaluate cartilage extent. Measurements were performed on the sagittal slice exhibiting the maximum cartilage length, according to Dejour et al. (34).

- PL was defined as the greatest length of the patellar cartilage in the sagittal position.
- TL was defined as the greatest length of the femoral cartilage in the sagittal position (Figure 3D).

P-PTA and Q-PA were measured on sagittal T1-weighted images. The mid-sagittal slice that provided the clearest simultaneous view of the patella, patellar tendon, and tibial tuberosity was selected for measurement.

- P-PTA was defined by Aksahin et al. (26) as the angle between the line connecting the lower pole of the patella to the tibial tuberosity and the line connecting the upper and lower poles of the patella (Figure 3F).
- Q-PA was defined as the angle formed between the line passing behind the deepest fiber of the quadriceps tendon and the line connecting the upper pole of the patella (Figure 3G).

### 2.4 Statistical analysis

The consistency of repeated measurements between two independent observers and within a single observer was evaluated using the intraclass correlation coefficient (ICC). An ICC of >0.75 indicated a high degree of consistency in the measurement results (35). The Kolmogorov–Smirnov test, which is particularly suitable for large sample sizes (*n* > 50) and demonstrates robust power in such contexts, was selected to assess normality. Therefore, an independent samples *t*-test was used for between-group comparisons. Cohen st power in such coto assess the effect size, which was interpreted according to Cohens the effect size, w (|*d*| = 0.2), medium (|*d*|= 0.5), and large (|*d*| = 0.8) (36). For the data that did not follow a normal distribution, the Mann–Whitney U test was employed. The effect size was estimated using r value, with the following conventional thresholds proposed by Cohen: small (|*r*| = 0.1), medium (|*r*| = 0.3), and large (|*r*| = 0.5). A Cohen’s *d* value > 0.8 or *r* > 0.5 was considered a large effect.

Univariate and multivariate logistic regression analyses were performed to assess the associations between the calculated ratios and the presence of mild CMP. Ratios with a significance level of *P* < 0.1 in the univariate analysis were included in the multivariate model, which was further adjusted for covariates including sex, age, affected side (left/right knee), height, weight, and BMI. A receiver operating characteristic analysis was performed for all measurements and ratios, and a function graph was plotted between the true-positive rate (i.e., sensitivity) and false-positive rate (i.e., 1–specificity). The AUC is a key indicator for evaluating predictive performance. AUC values range from 0.5 (no diagnostic value) to 1.0 (perfect diagnostic ability). When the AUC value exceeds 0.7, the diagnostic test is considered clinically significant. Youden’s J statistic was calculated to determine the optimal cutoff value, distinguishing the presence and absence of mild CMP based on each pair of sensitivity/specificity values (J = sensitivity + specificity–1). The AUC, positive predictive value (PPV), and negative predictive value (NPV) was calculated for each indicator. The predictive strength of each anatomical ratio for mild CMP was evaluated using statistical indicators such as the odds ratio (OR), 95% confidence interval, and *P*-value. Because TSA of >145°, LTI of <17°, and TD of <4 mm have been described as pathological thresholds (14, 37, 38), these values were included in this comparison. All analyses were conducted using SPSS 26.0 (IBM Corp., Armonk, NY, United States).

## 3 Results

The demographic characteristics of the patients included in this study are shown in Table 1. There was no significant difference in demographics between the two groups.

**TABLE 1:** Patient demographics.

Characteristics	Control group	Mild CMP group	χ ^2^/*t*	*P*-value
Number of knees	100	100	0.000	0.999
Minimum age (years)	15	15	–	–
Maximum age (years)	40	40	–	–
Mean age (years)	27.08 ± 6.74	27.01 ± 6.74	0.073	0.942
Male/female	42/58	42/58	0.000	0.999
Right knee/left knee	55/45	51/49	0.321	0.671
Minimum height (m)	1.55	1.56	–	–
Maximum height (m)	1.85	1.85	–	–
Mean height (m)	1.69 ± 0.09	1.70 ± 0.09	0.522	0.602
Minimum weight (kg)	44.93	46.89	–	–
Maximum weight (kg)	95.74	93.56	–	–
Mean weight (kg)	72.19 ± 12.70	73.50 ± 12.67	0.727	0.468
Minimum BMI (kg/m^2^)	17.99	18.12	–	–
Maximum BMI (kg/m^2^)	29.22	29.64	–	–
Mean BMI (kg/m^2^)	25.09 ± 3.23	25.34 ± 3.17	0.544	0.587

CMP, chondromalacia patella; BMI, body mass index.

The ICC values for intra-rater and inter-rater reliability are shown in Table 2. The intra-rater and inter-rater reliability ranged from 0.96 to 0.99 and from 0.90 to 0.96, respectively, indicating that all measurements results had moderate to high reliability.

**TABLE 2:** Intrarater and interrater reliability for all measurements.^a,b^

	Intrarater	Interrater
TSA	0.98 (0.93–0.99)	0.93 (0.91–0.95)
LTI	0.99 (0.96–0.99)	0.94 (0.92–0.96)
TD	0.96 (0.91–0.99)	0.9 (0.86–0.92)
TW	0.98 (0.96–0.99)	0.96 (0.94–0.97)
PW	0.98 (0.95–0.99)	0.94 (0.93–0.96)
PL	0.97 (0.91–0.99)	0.91 (0.88–0.93)
TL	0.97 (0.9–0.99)	0.9 (0.87–0.92)
P-PTA	0.97 (0.93–0.99)	0.94 (0.92–0.96)
Q-PA	0.98 (0.88–0.99)	0.96 (0.95–0.97)

*^a^*The data are presented as the intraclass correlation coefficient (presented outside the brackets) with the 95% confidence interval (presented inside the brackets).

*^b^*TSA, trochlear sulcus angle; LTI, lateral trochlear inclination; TD, trochlear depth; TL, trochlear length; PL, patellar length; TW, trochlear width; PW, patellar width; P-PTA, patella-patellar tendon angle; Q-PA, quadriceps-patellar tendon angle.

Several parameters and ratios differed significantly between the control and mild CMP groups. These included trochlear morphological parameters (TSA, LTI, TD), patellofemoral engagement parameters (TW, PW, P-PTA), and the composite ratios (TSA/TW, TSA/PW, TSA/P-PTA). Among these, the TSA was greater in the mild CMP group than in the control group, with Cohen’s d indicating a significant effect size. Large effect sizes (|Cohen’s *d*| > 0.8) were observed for TW, PW, and TSA/TW (Table 3).

**TABLE 3:** Comparison of all measurements and ratios between control and mild CMP.^c,d^

	Control group (*n* = 100 knees)	Mild CMP group (*n* = 100 knees)	Mean/Median difference (95% CI)	*P-*value	Effect size (Cohen’s *d*/*r*-value)
TSA (°)^a^	139.23 ± 4.32	140.46 ± 4.17	–1.23 (–2.41 to –0.05)	**0.04**	–0.29
LTI (°)^a^	19.90 ± 3.37	18.87 ± 3.10	1.03 (0.12–1.93)	**0.03**	0.32
TD (mm)^a^	5.80 ± 1.14	5.37 ± 1.11	0.43 (0.11–0.74)	**0.01**	0.38
TW (mm)^a^	36.46 ± 2.66	33.39 ± 2.04	3.06 (2.40–3.72)	**< 0.01**	**1.29**
PW (mm)^a^	37.96 ± 2.74	35.44 ± 2.23	2.52 (1.82–3.21)	**< 0.01**	**1.01**
TL (mm)^a^	14.20 ± 1.78	14.19 ± 3.02	0.01 (–0.68 to 0.70)	0.99	0.00
PL (mm)^a^	28.13 ± 2.05	27.76 ± 1.85	0.36 (–0.18 to 0.91)	0.19	0.19
P-PTA (°)^a^	143.94 ± 4.20	142.40 ± 2.20	1.55 (0.61–2.48)	**0.01**	0.46
Q-PA (°)^a^	37.93 ± 5.71	37.34 ± 6.33	0.59 (–1.09 to 2.27)	0.49	0.10
TSA/TW^a^	3.84 ± 0.34	4.22 ± 0.32	–0.38 (–0.47 to –0.29)	**< 0.01**	**–1.16**
TSA/PW^b^	3.69 (3.44–3.88)	3.96 (3.74–4.09)	–0.27 (–0.38 to –0.21)	**< 0.01**	0.45
TSA/TL^b^	9.81 (8.99–10.42)	9.53 (8.39–11.27)	0.28 (–1.08 to 0.12)	0.81	0.02
TSA/PL^b^	4.98 (4.66–5.18)	5.03 (4.75–5.25)	–0.06 (-0.22 to 0.01)	0.07	0.13
TSA/P-PTA^a^	0.97 ± 0.04	0.99 ± 0.03	–0.02 (–0.03 to –0.01)	**< 0.01**	–0.52
TSA/Q-PA^b^	3.65 (3.27–3.93)	3.76 (3.40–4.23)	–0.11 (–0.30 to 0.10)	0.35	0.07

*^a^*The Kolmogorov–Smirnov test revealed that the date in both the mild CMP group and the control group followed a normal distribution.

*^b^*The Kolmogorov–Smirnov test revealed that the date in one or both groups did not follow a normal distribution.

*^c^*Data are reported as mean (standard deviation) for normally distributed variables or median (interquartile range) for non-normally distributed variables. Effect size was measured with Cohen’s *d* statistic for parametric tests, while r value was used for non-parametric tests. Boldface text indicates statistical significance (*P* < 0.05) or large effect size (|Cohen’s *d*| > 0.80; |*r*| > 0.50).

*^d^*CI, Confidence Interval; CMP, chondromalacia patella; TSA, trochlear sulcus angle; LTI, lateral trochlear inclination; TD, trochlear depth; TL, trochlear length; PL, patellar length; TW, trochlear width; PW, patellar width; P-PTA, patella-patellar tendon angle; Q-PA, quadriceps-patellar tendon angle.

Univariate logistic regression analysis identified ratios significantly associated with mild CMP. Specifically, the ratios TSA/TW (OR = 39.61, 95% CI: 12.47–125.89, *P* < 0.01), TSA/PW (OR = 32.25, 95% CI: 9.60–108.42, *P* < 0.01), and TSA/P-PTA (OR = 15.03, 95% CI: 7.14–25.47, *P* < 0.01) demonstrated strong predictive effects. The ratio TSA/PL also approached significance (OR = 1.92, 95% CI: 0.95–3.90, *P* = 0.07). These ratios, along with the covariates sex, age, affected side, height, weight, and BMI, were included in the multivariate logistic regression model. After adjustment, only TSA/TW remained an independent and significant predictor of mild CMP (aOR = 55.45, 95% CI: 8.08–380.36, *P* < 0.01) (Table 4).

**TABLE 4:** Univariate and multivariate logistic regression analyses of ratios for diagnosing mild CMP.^a,b^

	Univariate analysis		Multivariate analysis	
Ratios	OR (95% CI)	*P*-value	aOR (95% CI)	*P*-value
TSA/TW	39.61 (12.47–125.89)	<0.01	55.45 (8.08–380.36)	<0.01
TSA/PW	32.25 (9.60–108.42)	<0.01	4.64 (0.62–34.93)	0.14
TSA/TL	1.11 (0.97–1.26)	0.14	–	–
TSA/PL	1.92 (0.95–3.90)	0.07	0.49 (0.18–1.30)	0.15
TSA/P-PTA	15.03 (7.14–25.47)	<0.01	1.22 (0.82–1.81)	0.43
TSA/Q-PA	1.24 (0.83–1.84)	0.29	–	–

*^a^*Ratios with a *P*−value < 0.1 in the univariate analysis were included in the multivariate model, which was additionally adjusted for sex, age, affected side (left/right knee), height, weight, and BMI. Results are presented as odds ratios (OR) or adjusted odds ratios (aOR) with their corresponding 95% confidence intervals (CI).

*^b^*TSA, trochlear sulcus angle; LTI, lateral trochlear inclination; TD, trochlear depth; TL, trochlear length; PL, patellar length; TW, trochlear width; PW, patellar width; P-PTA, patella-patellar tendon angle; Q-PA, quadriceps-patellar tendon angle.

**FIGURE 4:**
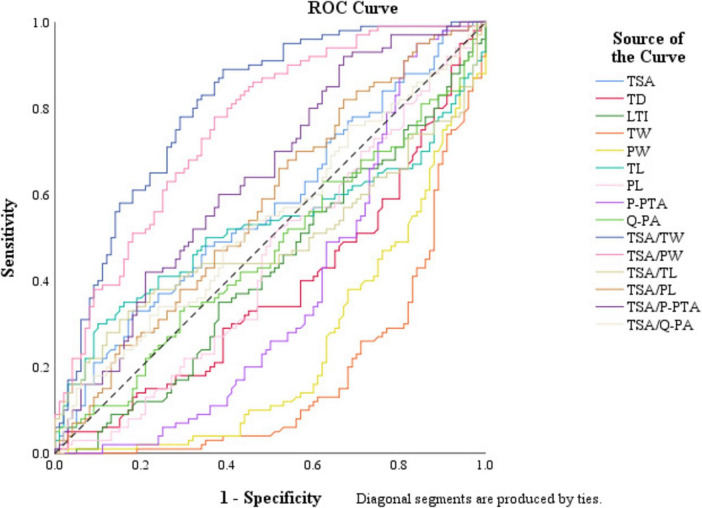
Receiver operating characteristic (ROC) curves for all measurements and ratios in diagnosing mild chondromalacia patellae. TSA, trochlear sulcus angle; LTI, lateral trochlear inclination; TD, trochlear depth; TL, trochlear length; PL, patellar length; TW, trochlear width; PW, patellar width; P-PTA, patella-patellar tendon angle; Q-PA, quadriceps-patellar tendon angle.

A receiver operating characteristic analysis was performed for all measurements and ratios (Figure 4). Youden’s J statistic was used to calculate the AUC, PPV, and NPV for each ratio. The AUC for all measured values and ratios ranged from 0.18 to 0.80. The AUC for both TSA/TW and TSA/PW reached 0.7 (indicating that these are valuable diagnostic tests), with TSA/TW having the highest AUC. The AUCs of TSA, TL, TSA/PL, TSA/PPTA, and TSA/QPA were all > 0.5 (indicating moderate or weak predictive ability) (Table 5).

**TABLE 5:** AUC and Youden’s J values for all measurements and ratios.^a,b^

	AUC (95% CI)	Youden’s J	PPV, %	NPV, %
TSA	0.57 (0.49–0.65)	0.15	64.7%	55%
LTI	0.43 (0.35–0.51)	0	0%	50%
TD	0.38 (0.3–0.46)	0.02	62.5%	50.5%
TW	0.18 (0.12–0.24)	0	0%	50%
PW	0.24 (0.18–0.31)	0	0%	50%
TL	0.52 (0.44–0.61)	0.20	75%	56.3%
PL	0.45 (0.37–0.53)	0.01	50.3%	100%
P-PTA	0.37 (0.29–0.45)	0.11	53.1%	76.2%
Q-PA	0.47 (0.39–0.55)	0.05	85.7%	51.3%
TSA/TW	**0.80 (0.74–0.87)**	0.50	69.5%	84.7%
TSA/PW	**0.76 (0.69–0.83)**	0.41	67.8%	74.1%
TSA/TL	0.49 (0.41–0.57)	0.16	70.0%	55.0%
TSA/PL	0.57 (0.50–0.65)	0.15	55.0%	64.7%
TSA/P-PTA	0.64 (0.57–0.72)	0.25	57.9%	80.5%
TSA/Q-PA	0.54 (0.46–0.62)	0.10	58.6%	53.5%

*^a^*Boldface text indicates an AUC of > 0.70.

*^b^*AUC, area under the curve; CI, confidence interval; PPV, positive predictive value; NPV, negative predictive value; TSA, trochlear sulcus angle; LTI, lateral trochlear inclination; TD, trochlear depth; TL, trochlear length; PL, patellar length.; TW, trochlear width; PW, patellar width; P-PTA, patella-patellar tendon angle; Q-PA, quadriceps-patellar tendon angle.

The ability of TSA, LTI, TD, and the six anatomical ratios to predict mild CMP was evaluated. When TSA was >145°, LTI was <17°, and TD was < 4mm, the predicted ORs for mild CMP were 2.19, 1.69, and 1.74, respectively (*P* > 0.05 for all). Among the six ratios studied, TSA/TW, TSA/PW, and TSA/PPTA produced the highest ORs (12.66, 6.04, and 5.66, respectively). Among the six ratios analyzed, only TSA/QPA showed no statistically significant results (Table 6).

**TABLE 6:** Comparison of predictive ability of ratios.^a,b^

	Cutoff value	Odds ratio (95% CI)	*P*-value	Sensitivity, %	Specificity, %
TSA (°)	>145	2.19 (0.89–5.38)	0.126	16.0%	92.0%
LTI (°)	<17	1.69 (0.85–3.22)	0.182	28.0%	81.0%
TD (mm)	< 4	1.74 (0.61–4.99)	0.435	10.0%	94.0%
Ratios					
TSA/TW	>3.9	12.66 (6.01–26.64)	<0.001	89.0%	61.0%
TSA/PW	>3.8	6.04 (3.24–11.26)	<0.001	78.0%	63.0%
TSA/TL	>11.3	2.85 (1.36–6.00)	0.008	28.0%	88.0%
TSA/PL	>4.8	2.24 (1.16–4.34)	0.023	82.0%	33.0%
TSA/P-PTA	>1.0	5.66 (2.46–13.04)	<0.001	92.0%	33.0%
TSA/Q-PA	>4.0	1.63 (0.88–3.03)	0.160	34.0%	76.0%

*^a^*An OR of > 1 signified that mild chondromalacia patella was more likely in the mild chondromalacia patella group than in the control group, with higher values indicating stronger associations.

*^b^*TSA, trochlear sulcus angle; LTI, lateral trochlear inclination; TD, trochlear depth; TL, trochlear length; PL, patellar length; TW, trochlear width; PW, patellar width; P-PTA, patella-patellar tendon angle; Q-PA, quadriceps-patellar tendon angle.

## 4 Discussion

In this study involving the knee joints of 100 healthy volunteers and 100 patients with mild CMP, this is the first study to propose TSA/TW and TSA/PW as composite ratios for early identification mild chondromalacia patellae (CMP), providing a more accurate scientific basis for early diagnosis and treatment of CMP. This distinction from previous TSA-only studies significantly enhances sensitivity in diagnosing mild CMP. These ratios may play a crucial role in preventing the progression of advanced osteoarthritis.

The significance and effect size of TW and PW (Cohen’s *d* values of 1.29 and 1.01, respectively) indicate that changes in patellar structure may be related to the development of CMP. Previous studies have shown that reductions in TW and PW may lead to instability of the patellofemoral joint, increase the joint surface pressure, and subsequently cause symptoms of cartilage injury and CMP (37, 39, 40). In terms of the anatomical structure between the patella and the femoral trochlear groove, TW and PW are parameters reflecting patellar lateral displacement, and both are of great significance for maintaining patellar localization and movement within the femoral trochlea (33, 41–43). If the patellar position is abnormal or lateral displacement occurs, TW may increase, according to anatomical and biomechanical principles, changes in TW—whether increases or decreases—can indicate abnormal patellar tracking and thus increasing the risk of CMP. Therefore, TW and PW can serve as important parameters for evaluating the risk of CMP.

Because of the unique anatomical features of the patellofemoral joint, the cartilage is extremely susceptible to damage. The angle of the femoral trochlear groove is one of the factors determining the development of the femoral trochlea. Femoral trochlear dysplasia, characterized by a shallow trochlear groove, is accompanied by lateral patellar instability (27). In this study, the AUC of TSA was the highest among all trochlear morphologic and patellofemoral joint parameters. Numerous other studies have also explored the relationship between trochlear dysplasia and mild CMP (4). Endo et al. (44) found no correlation between CMP and TD. Damgac et al. (10) reported that mild CMP was not associated with TSA, whereas patients with severe CMP had a larger TSA than participants in the control group. Although the TSA has been shown to be associated with CMP, its limitation is that a pathological TSA, as a fixed value, has lower sensitivity in diagnosing mild CMP and may not be applicable to all patients. To overcome this limitation, we established six ratios with TSA as the numerator and patellofemoral joint parameters as the denominator. The AUC of TSA/TW and TSA/PW both reached 0.7, indicating certain diagnostic value.

Because of the unique anatomy of the patellofemoral joint, its articular cartilage is particularly vulnerable to mechanical overload. Trochlear morphology plays a central role in patellar tracking, and trochlear dysplasia—characterized by a shallow groove and increased sulcus angle—is closely associated with lateral patellar instability. In the present study, TSA demonstrated the highest AUC among individual trochlear and patellofemoral parameters, supporting its relevance in early CMP. However, prior studies have yielded inconsistent results regarding the relationship between trochlear dysplasia and mild CMP. Endo et al. reported no correlation between TD and CMP, while Damgac et al. found that TSA was associated only with severe, but not mild, CMP. These findings suggest that although TSA reflects underlying trochlear morphology, its use as a fixed pathological threshold may lack sufficient sensitivity for detecting early cartilage changes. To address this limitation, we constructed composite ratios incorporating TSA with patellofemoral engagement parameters. TSA/TW and TSA/PW achieved AUC values ≥ 0.7, indicating improved diagnostic performance compared with TSA alone. Notably, ratios involving TW and PW outperformed those incorporating PL and TL. TW and PW reflect axial patellar positioning relative to the trochlea, whereas PL and TL primarily assess sagittal alignment and patellar height. This distinction suggests that axial engagement between the patella and trochlea may play a more critical role in early cartilage injury than sagittal alignment alone. Mechanistically, trochlear dysplasia increases TSA by reducing the concavity and depth of the trochlear groove, thereby compromising lateral containment. When combined with reduced axial engagement (narrower TW or PW), this imbalance may exacerbate patellofemoral instability and increase focal cartilage stress. Accordingly, larger TSA/TW and TSA/PW ratios were associated with a higher likelihood of mild CMP, highlighting the importance of integrating morphological and engagement parameters to better capture early biomechanical dysfunction.

Ratios involving TW and PW are more predictive than ratios involving PL and TL. TW and PW are indicators of axial patellar displacement relative to the trochlea (15), while PL and TL are assessments of sagittal patellar and femoral trochlear function and can serve as supplementary tools for evaluating patellar height (45). Based on these findings, the engagement between the patella and trochlea in the axial plane may play a more important role in patellar stability. It is clear that larger TSA/TW and TSA/PW ratios are associated with a higher likelihood of patellofemoral cartilage defects and patellofemoral joint instability. Dysplasia of the femoral trochlea manifests as loss of the concave anatomical structure and depth of the femoral trochlear groove, leading to an increase in TSA, which is a known risk factor. Patellofemoral instability is also considered to induce cartilage injury (46).

The vastus medialis oblique is one of the muscle bundles that contributes most to resisting lateral displacement of the patella and has a dynamic constraint effect (47, 48). Increase in the TSA/TW and TSA/PW ratios indicates that the patella can reach lateral dislocation at a shorter distance. This may be due to underdeveloped medial soft tissues such as the vastus medialis oblique and medial patellofemoral ligament, which result in weak constraint force. At the same time, a shallow pulley can further reduce the constraint of the patellar cartilage, making dislocation more likely to occur. From a biomechanical perspective, an increase in lateral mobility of the patella leads to a disproportionate load distribution in the patellofemoral joint. When the quadriceps muscle contracts, the contact area between the patellar cartilage and the trochlear cartilage on the rotation axis decreases, resulting in higher contact pressure and acceleration of cartilage damage. Therefore, patients with increased TSA/TW and TSA/PW also have an increased risk of CMP.

Fan et al. (49) observed that an increased TSA induced patellofemoral instability, potentially leading to thinning of the lateral trochlear cartilage. Davies Tuck et al. (50) also found that an increased TSA was associated with an increase in the medial patellar cartilage volume and that shallower grooves may be a determining factor in protecting the medial compartment from the influence of degenerative processes. According to Aksahin et al. (26), patients with grade 3 and 4 CMP have sagittal patellar tilt (reduced PPTA) in the patellofemoral joint, with the tilt concentrated in the upper and lower areas of the patellar cartilage. No significant changes were found in the PTA of patients with intermediate cartilage lesions. Therefore, there was no consistency between axial (increased TSA) and sagittal (decreased PPTA) misalignment in early identification the location of patellofemoral cartilage defects. This discrepancy between axial and sagittal misalignment may explain the limited diagnostic utility of TSA/PPTA in early-stage CMP.

## 5 Limitations

The main limitation of this study is that the diagnosis of mild CMP was only validated through conventional MRI and T2 mapping techniques; the patients were not directly evaluated under arthroscopy to determine the presence or absence of this diagnosis. In addition, because the MRI scans were performed with the patients in the supine position rather than in a weight-bearing state, the ability to measure the dynamic changes in patellar position during weight-bearing was limited. Therefore, for measurement indices that may change with weight-bearing, such as PPTA and QPA, it is necessary to take this limitation into account. Finally, data on the patients’ socioeconomic status (work, sports life, etc.) and activity level could not be obtained.

## 6 Conclusion

The ratios of patellofemoral engagement (TSA/TW and TSA/PW) are more accurate in early identification the occurrence of mild CMP than is TSA alone, with TSA/TW having the strongest predictive ability. Each ratio takes the patient’s specific anatomical structure into account and can be measured accurately and reliably by clinical doctors. These ratios represent a new step toward overcoming the limitations of low sensitivity when using TSA alone to predict mild CMP.

## Data Availability

The data supporting the findings of this study are available from the corresponding author upon reasonable request.
